# The gut microbiota in osteoarthritis: where do we stand and what can we do?

**DOI:** 10.1186/s13075-021-02427-9

**Published:** 2021-01-27

**Authors:** Xiaoxia Hao, Xingru Shang, Jiawei Liu, Ruimin Chi, Jiaming Zhang, Tao Xu

**Affiliations:** 1 Department of Rehabilitation, Tongji Hospital, Tongji Medical College, Huazhong University of Science and Technology, 1095#, Jie-Fang Avenue, Qiaokou District, Wuhan, 430030 Hubei China; 2 Department of Orthopedics, Tongji Hospital, Tongji Medical College, Huazhong University of Science and Technology, 1095#, Jie-Fang Avenue, Qiaokou District, Wuhan, 430030 Hubei China; 3 Cancer Center, Union Hospital, Tongji Medical College, Huazhong University of Science and Technology, Wuhan, 430022 China

**Keywords:** Osteoarthritis, Gut microbiota, Gut dysbiosis, Inflammation, Exercise, Fecal microbiota transplantation

## Abstract

Osteoarthritis (OA) is one of the most frequent musculoskeletal diseases characterized by degeneration of articular cartilage, subchondral bone remodeling, and synovial membrane inflammation, which is a leading cause of global disability, morbidity, and decreased quality of life. Interpreting the potential mechanisms of OA pathogenesis is essential for developing novel prevention and disease-modifying therapeutic interventions. Gut microbiota is responsible for a series of metabolic, immunological, and structural and neurological functions, potentially elucidating the heterogeneity of OA phenotypes and individual features. In this narrative review, we summarized research evidence supporting the hypothesis of a “gut-joint axis” and the interaction between gut microbiota and the OA-relevant factors, including age, gender, genetics, metabolism, central nervous system, and joint injury, elucidating the underlying mechanisms of this intricate interaction. In the context, we also speculated the promising manipulation of gut microbiota in OA management, such as exercise and fecal microbiota transplantation (FMT), highlighting the clinical values of gut microbiota. Additionally, future research directions, such as more convincing studies by the interventions of gut microbiota, the gene regulation of host contributing to or attributed to the specific phenotypes of gut microbiota related to OA, and the relevance of distinct cell subgroups to gut microbiota, are expected. Moreover, gut microbiota is also the potential biomarker related to inflammation and gut dysbiosis that is able to predict OA progression and monitor the efficacy of therapeutic intervention.

## Introduction

Osteoarthritis (OA) is one of the most frequent musculoskeletal diseases characterized by degeneration of articular cartilage, subchondral bone remodeling, synovial membrane inflammation causing pain, morning stiffness, swelling, limited range of joint motion, and poor physical function, which is a leading cause of global disability, morbidity, and decreased quality of life [1]. With aging and increasing obesity population, the prevalence of osteoarthritis is rising, particularly since the mid-twentieth century, impacting 303 million individuals globally in 2017 and resulting in unsustainable clinical, humanistic, and economic burdens composed of medical care costs, lost wages, and depressed economic productivity [2–4].

OA is now recognized as a collection of multiple subgroups, each with specific pathophysiological and clinical features dependent on the risk factors involved, such as metabolic syndrome-associated osteoarthritis, post-traumatic osteoarthritis, and aging-associated osteoarthritis [5–7]. These risk factors independently or intricately contribute to a complex interaction between mechanical, biochemical, and cellular factors, leading to the pathogenesis of OA. Identifying phenotypes of patients may help to detect the disease at its early stage and could be used to guide clinical decision making and allow more effective and specific therapeutic interventions targeted toward individuals. As a result, it is meaningful to identify an underlying but important individual feature, gut microbiota, to better understand the heterogeneity of OA phenotypes.

*Gut microbiota*, defined as a collection of gut microbe populations, is responsible for a series of metabolic, immunological, structural, and neurological functions, such as maintenance of metabolic homeostasis, development and maturation of immune system, resistance to infections, and production of neurotransmitters [8]. The most representative bacterial phyla in gut microenvironment are *Firmicutes* and *Bacteroidetes*, followed by *Verrucomicrobia*, *Actinobacteria*, *Fusobacteria*, *Proteobacteria*, and *Cyanobacteria* [9]. Due to its incredible involvements, *microbial dysbiosis*, defined as an adverse alteration in the diversity, structure, or function of gut microbiota, contributes to diverse pathological states and diseases. Gut microbiota is involved in the initiation and progression of inflammation-driven diseases, and microbial dysbiosis has been emerged as a hidden risk factor inducing the production of proinflammatory cytokines and bacterial metabolites, which may boost the pathophysiological mechanisms of OA [10]. OA risk factors, such as aging, diet, and obesity, are shown to perturbate gut microbiota, while limited evidence supports the involvement of gut microbiota upon the mechanisms of these risk factors.

**Fig. 1:**
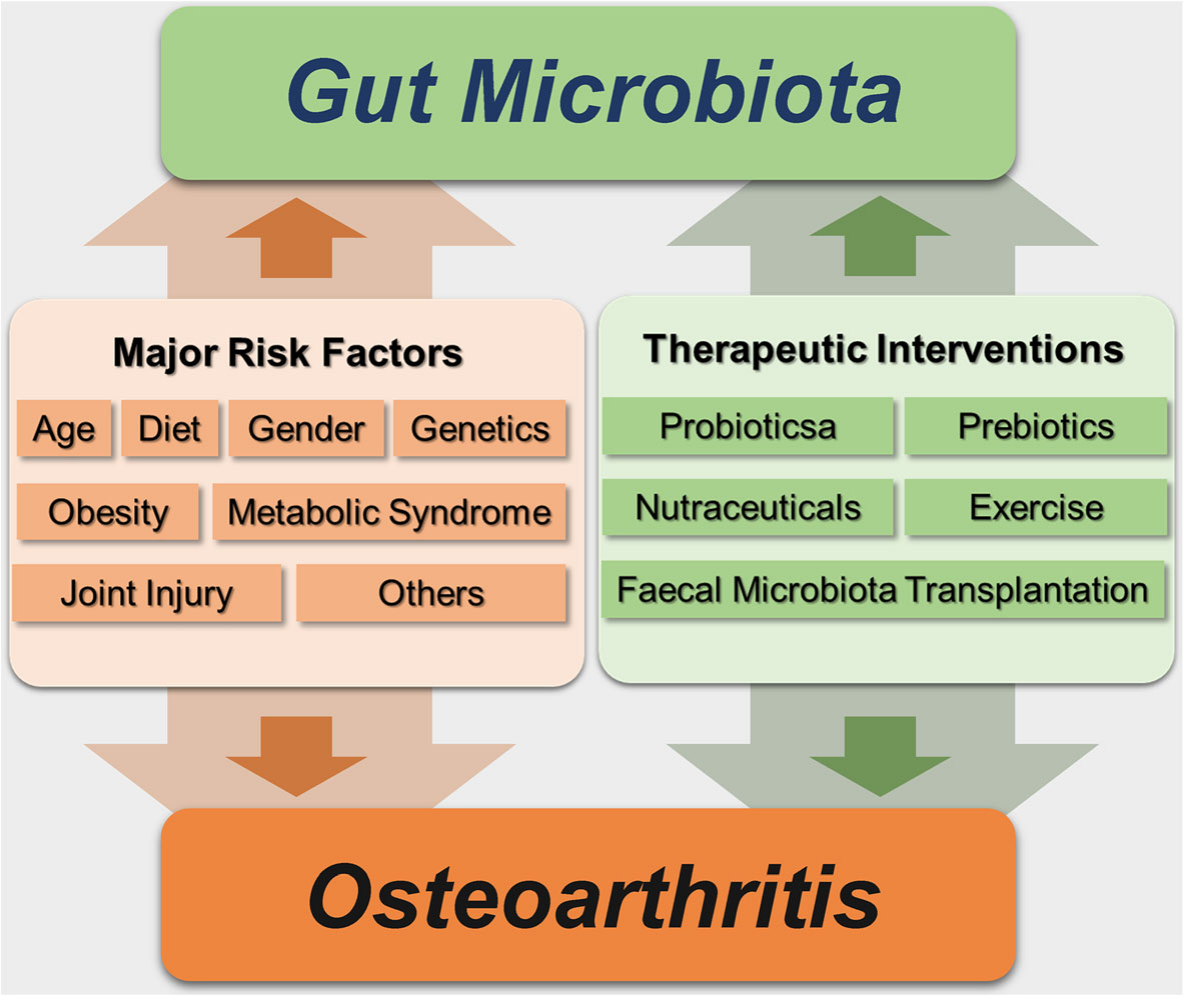
Relationship between the gut microbiota and osteoarthritis development. OA-relevant factors are involved in OA either directly, or via the modulation of gut microbiota. Several disease-modifying therapeutic approaches can relieve OA symptoms directly, or via altering the composition of gut microbiota to influence OA progression indirectly. OA, osteoarthritis

In this review, we summarized the evidence supporting the hypothesis of “gut-joint” axis and the interactions between gut microbiota and OA-relevant factors and assessed the potentials of microbiota-targeted therapies in OA management (Table 1, Fig. 1). Based on the current understanding of the crosstalk between gut microbiota and these factors, gut microbiota could be considered as an indispensable element that provides a unifying mechanism to explain the involvement of these individual-level risk factors in OA.

**Table 1:** Summary of studies supporting the contribution of gut microbiota to OA

Study	Study setting	Intervention	Duration	Key findings
Collins et al., 2015 [11]	HFS diet-induced obesity rat model	N/A	N/A	Greater Modified Mankin Scores, and higher level of serum LPS in obese animals. Lactobacillus species and Methanobrevibacter spp. abundance correlated with Mankin scores.
Guss et al., 2019 [12]	Toll-like receptor-5 deficient (TLR5KO) mice, HFD-induced obesity rat model	Tibial cyclic compressive loading	2 weeks or 6 weeks	After 2 weeks of loading, cartilage damage (OARSI score) was not different among groups. After 6 weeks of loading, HFD mice had increased load-induced cartilage damage and elevated serum inflammatory markers. TLR5KO mice is not sufficient to develop severe cartilage damage and treated with chronic antibiotics reduces OA severity. Each group had a distinct gut microbiome composition.
Ulici et al., 2018 [13]	DMM -induced OA mice model	N/A	N/A	Reduced maximum ACS scores in GF mice compared to SPF mice. Differences in abundance of microbes detected in SPF mice with high or low maximum ACS scores.
Lei et al., 2017 [14]	Patients with symptomatic knee OA (*n* = 537)	Skimmed milk containing either probiotic LcS or placebo daily	6 months	After 6 months of treatment, WOMAC and VAS scores were significantly decreased in the LcS groups of patients compared to the placebo group. Serum levels of hs-CRP were also significantly lower in patients receiving LcS than placebo.
So et al., 2011 [15]	MIA-induced OA rat model	LcS ± CII/Gln	10 weeks	Oral administration of LcS together with CII and Gln more effectively reduced pain, cartilage destruction, and lymphocyte infiltration than the treatment of Gln or LcS alone.
Sim et al., 2018 [16]	MIA-induced OA rat model	ID-CBT5101 (tyndallized *Clostridium butyricum*)	6 weeks	ID-CBT5101 treatments effectively preserved the knee cartilage and synovial membrane and significantly decreased the amount of fibrous tissue.
Kwon et al., 2018 [17]	MIA-induced OA rat model	Probiotic complex, rosavin, and zinc	Not indicated	The combination improved OA pain levels by preventing cartilage damage, reduced the expression of proinflammatory cytokines and catabolic factors, and increased the production of anti-inflammatory cytokines as well as the anabolic factor.
Schott et al., 2018 [18]	DMM -induced OA mice model of HFD-induced obesity and in lean mice	Oligofructose (prebiotic)	12 weeks	Prebiotic supplementation reduced OA severity in obese mice. *Bifidobacterium pseudolongum* abundance increased with prebiotic treatment and its levels were inversely associated with OA severity, systemic, and colon inflammation.
Coulson et al., 2013 [19]	Patients diagnosed with knee OA (*n* = 38)	GLM extract or GS	12 weeks	Both GLM and GS treatment reduced OA symptoms. In both groups there was a trend toward a decrease in Clostridium and Staphylococcus species and increase in Lactobacillus, Streptococcus and Eubacterium species.
Rios et al., 2019 [20]	HFS diet-induced obesity rat model	Prebiotic fiber supplementation ± HFS diet ± aerobic exercise	12 weeks	Prebiotic fibers, exercise, and the combination of both prevented knee joint damage. The combinative treatments increased the abundance of Bifidobacterium and Roseburia and decreased *Clostridium leptum* and *Akkermansia muciniphila*.
Huang et al., 2020 [21]	MLI-induced OA germ-free mice model	FMT (fecal samples collected from human healthy controls, knee OA without metabolic syndrome and knee OA with metabolic syndrome)	10 weeks	OA severity was minimal in GF mice following MLI. Compared with the other groups, transplantation with the knee OA with metabolic syndrome groups, microbiome was associated with higher systemic levels of inflammatory biomarkers, higher gut permeability and worse OA severity. A greater abundance of Fusobacterium and Faecalibaterium and lesser abundance of Ruminococcaceae in transplanted mice were consistently correlated with OA severity and systemic biomarkers concentrations.

*OA* osteoarthritis, *HFS* high-fat/high-sucrose, *HFD* high-fat diet, *LPS* lipopolysaccharides, *OARSI* Osteoarthritis Research Society International, *DMM* destabilization of the medial meniscus, *ACS* articular cartilage structure, *GF* germ-free, *SPF* specific pathogen-free, *LcS Lactobacillus casei Shirota*, *WOMAC* Western Ontario and McMaster Universities Osteoarthritis Index, *VAS* visual analog scale, *HS-CRP* high-sensitivity C-reactive protein, *MIA* monosodium iodoacetate, *CII* type II collagen, *Gln* glucosamine, *GLM* green-lipped mussel, *GS* glucosamine sulfate, *MLI* meniscal/ligamentous injury, *FMT* fecal microbiota transplantation

## Hypothesis of “gut-joint” axis in OA

The “gut-joint” axis is established on the possibility of the crosstalk between joint and gut. It is well accepted that gut microbiota have been shown to produce a wide range of molecules, including enzymes, short-chain fatty acids (SCFAs), and metabolites. As a result, these bacterially produced proinflammatory metabolites, such as lipopolysaccharide (LPS), make their way from the “leaky gut” to the systemic circulation and induce systematic inflammation. Due to the elevation of LPS levels in association with obesity and metabolic syndrome which are the highly relevant risks to OA, it is easy to speculate the microbiota involvement in OA at least, by LPS-induced low-grade inflammation, metabolic endotoxemia, macrophage activation, and joint damage. Indeed, Huang et al. found that increased levels of lipopolysaccharide (LPS) and LPS-binding protein (LBP) were associated with knee osteophyte severity and abundance of activated macrophages in the synovium [22]. Also, monitoring of circulating LPS concentrations could provide a new means to diagnose and treat specific phenotypes of OA [23]. Furthermore, a recent interesting study performed by Christopher et al. identified a microbial DNA signature, for the first time, in human and mouse cartilage, the alternation of which is associated with the development and progression of human OA [24]. These findings suggest a potential involvement of microbiota by direct inoculation or the transportation of immune cells, while it is still a puzzle the detailed role of this specific bacterial DNA in cartilage tissue in OA pathogenesis.

## The interactions between gut microbiota and OA-relevant factors

Accumulating evidence reveals that gut microbiota could be re-sharped by some OA-relevant factors, such as aging, gender, diet, and obesity, parallelly boosting the systematic inflammation, suggesting the possibility of the microbe involvement in OA, while limited convincing studies have validated this speculation by the interventions on gut microbiota. Still, the knowledge of the interactions between the OA-relevant factors and gut microbiota adds a novel layer of our understanding of the complexity of OA pathogenesis and also provides a new perspective on OA investigation.

### Age

Aging has been shown to be associated with an increased incidence of osteoarthritis [25]. Previous studies have illuminated several potential mechanisms by which the aging-associated changes in articular tissues promote the progression of OA, such as pervasive aging-relevant chronic low-grade inflammation (also known as “inflammaging”), cellular senescence, mitochondrial dysfunction and oxidative stress, dysfunctional energy metabolism, and alternated mechanical properties extracellular matrix attributed to the accumulation of advanced glycation end products (AGEs) [26, 27].

In recent years, researchers are becoming increasingly interested in elucidating aging-related differences in the gut microbiota among elderly adults and young individuals to improve the understanding of aging-related mechanisms and discover novel therapies. Aging-related alterations in gut physiology, such as degenerative changes in enteric nervous system, gastric hypochlorhydria, drug perturbation, and motility disorders, have significant impacts on the diversity, composition, and functional features of gut microbiota [28]. The aging-related changes of gut microbiota were characterized by reduced diversity, decreased abundance of dominant species, increased abundance of subdominant species, and a swift of increased proteolytic and decreased saccharolytic bacteria [29–31]. For instance, Biagi et al. observed that the structure of the gut microbiota of centenarians differs significantly from that of non-centenarian adults, characterized by an enrichment in *Proteobacteria*, a phylum including many potentially pathogenic bacteria. Furthermore, these alterations in centenarians are associated with increased levels of proinflammatory markers, suggesting the potential role of gut microbiota in this pervasive systematic inflammation [32]. Given that aging is a persistent factor, the aging-associated differences in gut microbiota and relevant inflammatory status may be an important determinant of onset and progression of OA, while the intervention of aging-relevant microbiota is still needed to validate the involvement of gut microbiota in aging-relevant OA phenotypes.

### Gender

It is a striking observation that females are at greater risk for developing knee, hip, and hand OA compared to males and tend to be more severe [33]. The underlying mechanisms behind the increased incidence of OA with sex difference remains to be unclear, while this higher prevalence of OA observed in women at the time of menopause has been attributed to the hypothesis that hormonal factors, such as estrogens, play a role in the development of OA, for instance, estrogens increase the sensitivity to inflammatory stimuli and responses in female [34]. Moreover, some possible conjectures also have been proposed for explaining the discrepancy of gender-dependent influence in OA, such as sex differences in gut microbiota composition [35].

Comparing the gut microbiota composition of 341 female with 348 male mice, Elin et al. showed a decreased relative abundance of *Porphyromonaceae* and *Rikenella* and a higher abundance of *Ruminococcus*, *Coprococcus*, and *Dorea* in male mice, while a higher presence of *Allobaculum*, *Anaeroplasma*, and *Lactobacillaceae* and *Veilonellaceae* in female mice, demonstrating that sex differences exist in microbiota composition [36]. A recent clinical study was conducted in a subgroup of 75 patients (39 men and 36 women), who had similar dietary background and matched by age, to analyze differences in fecal samples intestinal microbiota composition by 16S DNA sequencing, observing that women had a higher abundance of *Bacteroides* and *Bilophila* compared to men while men present higher abundance of *Veillonella* and *Methanobrevibacter* [37].

The different composition of gut microbiota between males and females may be mediated by sex hormones. Although no study has validated that these gender-relevant difference of gut microbiota contributes to the higher OA prevalence of female individuals, Li et al. built the link between sex steroid, bone loss, and gut microbiota. Their study showed that sex steroid deficiency led to the increase of gut permeability and sex steroid deficiency-induced bone loss is gut microbiota-dependent [38]. Given that subchondral bone loss is a phenotype of OA, this evidence gives a clue that gut microbiota might be involved in OA subchondral bone loss and the gender divergence in gut microbiota might have an indispensable role in the definition of gender differences in the prevalence of OA.

### Genetics

Genetic factor is responsible for 60% of hand and hip OA and 40% of knee OA [39]. An increasing number of susceptibility loci, such as insulin-like growth factor 1 gene (*IGF1*), growth differentiation factor 5 gene (*GDF5*), and vitamin D receptor gene (*VDR*), are proven to contribute to genetic predisposition of OA onset [40, 41].

Solovieva et al. found that *VDR* gene polymorphisms play a role in the etiology of symmetrical hand OA in Finnish population [42]. Moreover, the effect of *VDR* polymorphisms on the risk of OA may be modified by daily calcium intake. Interestingly, the influence of *VDR* gene variation on gut microbiota was also demonstrated in a genome-wide association analysis (GWAS) study performed by Wang et al. [43]. Still, no evidence supports the role of genetic susceptibility of *VDR* gene in the interaction between host and gut microbiota on OA onset and progression while we believe that this critical gene in bone and joint health may contribute to the individual difference of OA patients by the involvement in host-gut interaction. In the context, elucidating the association between gut microbiota and genetic factors is an important contribution to our comprehension of how these manipulations of the bacteria or its metabolites affect bone and cartilage growth in individual levels.

### Diet

Several dietary factors have been reported to be involved in the pathophysiology of OA, such as polyunsaturated fatty acids, antioxidants, and amino acids [44, 45]. Baker et al. found a positive connection between the n-6 polyunsaturated fatty acid (PUFA), arachidonic acid (AA), and synovitis but an inverse relation between total plasma n-3 PUFA, docosahexaenoic acid (DHA), and patellofemoral cartilage loss, suggesting that systemic levels of n-3 and n-6 PUFAs which are manipulated by diet may be associated with articular-cartilage composition and structural damage [46]. Previous studies have showed that the intake of dietary antioxidants such as vitamin C, D, and K, involved in regulating collagen formation, bone metabolism, and cartilage mineralization, may prevent the progression of OA [44]. Although the effects of dietary supplements on OA prevention are controversial, it is no doubt that these nutrients are indispensable and at least benefit metabolic health. In this context, the improved understanding of how these dietary factors contribute to the maintenance of joint fitness is helpful to interpret the association between OA and unfavorable diet and give promise to diet-based supplemental strategies.

Gut microbiota is highly shaped and modulated by the host’s dietary components [47]. Kaliannan et al. found that mice fed a diet high in n-6 PUFAs can increase the proportions of LPS-producing and/or proinflammatory bacteria including *Proteobacteria* and its members, and decrease levels of LPS-suppressing and/or anti-inflammatory bacterial groups, such as *Bifidobacterium*, *Lactobacillus*, *Clostridium*, and *Enterococcus faecium*, while transgenic conversion of tissue n-6 to n-3 fatty acids dramatically exhibited opposite effect, suggesting that the tissue n-6/n-3 PUFA ratio modifies gut microbiota profile composition and gut permeability, leading to differential inflammatory status and metabolic syndrome [48]. Moreover, recent evidence showed that vitamin D may influence disease risk by modifying the diversity of gut microbiota. The effects of vitamin D on *Bacteroides* phylum are not conclusive while some studies have reported that a low vitamin D diet or vitamin D receptor (VDR) knock-out results in a more inflammatory fecal microbiome characterized by an increased *Bacteroidetes* [49, 50]. Interestingly, very high-dose vitamin D diet is unexpectedly associated with an increase in *Bacteroidetes*, particularly of the orders *Bacteroidales* and *Flavobacteriale*, with a decrease in circulating vitamin D [51], suggesting that vitamin D may influence host systematic immunology by modifying the diversity of gut microbiota in a complex feedback loop. Vitamin D may play a role in maintaining the mucosal barrier integrity by upregulating the expression of tight junction and adherent junction proteins and suppressing epithelial cell apoptosis [52]. Also, dietary glutamine supplementation alters composition and metabolism of intestinal microbiota, inducing a shift in the *Firmicutes*/*Bacteroidetes* ratio and enhanced intestinal secretory IgA (SIgA) secretion [53]. Although these interesting findings indicate that dietary nutrients influence host physiological functions dependent on intestinal microbiota, limited evidence supports that dietary nutrients can modify the OA-relevant microbiota, as a result of which the link between diet, gut microbiota, and OA is still not convincing and needs more experimental validations.

### Obesity and metabolic syndrome

Obesity is an established risk factor for osteoarthritis not only in weight-bearing joints, such as knee and hip, but also in non-weight-bearing joints, such as hand and temporomandibular joints, indicating that obesity contribute to the systemic factors relevant to OA [54, 55]. Metabolic syndrome-associated osteoarthritis (Met-OA) is the phenotype of OA characterized by obesity, diabetes, dyslipidemia, and hypertension [56]. Present studies have demonstrated several mechanisms linking osteoarthritis to obesity and metabolic syndrome partially, such as insulin resistance, the potential implication of oxidized low-density lipoprotein (ox-LDL) on ectopic bone formation and synovium inflammation, and the potential involvement of gut microbiota [57].

The most accepted connection between OA and obesity and metabolic syndrome is the low-grade chronic inflammation partially induced by the elevated microbiota-derived proinflammatory metabolites and relevant gut microbiota components, such as LPS. Collins et al. found that systematic LPS concentration was associated *Lactobacillus* species abundance and increased joint damage was associated with the abundance of *Lactobacillus* and *Methanobrevibacter* species and body fat, but not body mass, in a high-fat/high-sucrose diet-induced obese rat model [11]. Another observation performed by Guss et al. in a load-induced model of OA with/without a high-fat diet in Toll-like receptor-5 deficient (TLR5KO) mice that spontaneously develop metabolic syndrome due to gut microbiota alterations, suggests that severe obesity and inflammation increased load-induced cartilage damage and the modification of metabolic syndrome-associated phylotypes of gut microbiota may contribute to development of cartilage pathology and subchondral bone morphology [12]. More importantly, the finding of Schott et al. suggests that oligofructose supplementation restored the lean gut microbiome in obese mice by supporting favorable *Bifidobacterium pseudolongum* and reduced OA progression, which sheds light to potential novel OA therapeutics involving strategic manipulation of specific microbial species inhabiting the intestinal space [18].

### Central nervous system (CNS)

It has been known for some time that the role of the central nervous system (CNS) is related to chronic pain in OA patients [58]. Progress of CNS theory in OA was accelerated in the early 1950s when neurophysiology was advancing [59]. So far, the new components of the CNS theory in OA pathophysiology include hypothalamic-pituitary (HPA) axis, nucleus tractus solitarus (NTS), hypothalamic suprachiasmatic nuclei (SCN), and other associated higher centers, and each with their own feedback circuits from the gut microbiota, OA joints, and cellular metabolism. Progression of OA is increasingly linked to dysregulation of central feedback circuits (e.g., HPA axis and NTS), which control circadian rhythm, gut microbiome, metabolism, and redox regulation [60].

Gut microbiota is regarded as one key element of the gut-brain axis in the CNS theory. Briefly, CNS modulates the gastrointestinal tract and enteric nervous system through sympathetic and parasympathetic axis while intestinal microbiota influence CNS function through vagal afferent nerves, immune system, HPA axis, and bacterially derived neurotransmitters [61, 62]. Additionally, due to the findings of Liang et al. that the absolute amount of fecal bacteria and the abundance of *Bacteroidetes* exhibited circadian rhythmicity, gut microbiota may communicate with host’s circadian clock to regulate cartilage homoeostasis [63, 64]. The interaction between CNS, gastrointestinal tract, and joint is an active area that remains to be investigated. Targeting gut microbiota to restore the balance of gut-brain axis might offer novel disease-modifying therapies for OA patients.

### Joint injury

Joint injury is a well-established risk factor for development of OA, including anterior cruciate ligament (ACL) rupture, meniscal tear, and intra-articular fracture. Nearly 12% of all OA cases may be due to initial trauma [65]. Therefore, a better understanding of mechanisms triggered by joint injury is beneficial to develop more targeted strategies for prevention and treatment of post-traumatic OA (PTOA). Accumulating evidence demonstrates that perpetuating inflammation response to joint injury plays a critical role in the progression of PTOA, including the production of inflammatory mediators, such as cytokines/chemokines and damage-associated molecular patterns (DAMPs), low-grade synovial immune infiltration, and innate inflammatory pathway activation involving multiple tissues [66].

Toll-like receptors (TLRs), most studied in PTOA among several receptors, are associated with proinflammatory innate immune response to injury through recognizing fragments of the cartilage extracellular matrix, microbiome-derived metabolites. Additionally, the combination of LPS and DAMPs resulting from joint damage, synergistically activates macrophages to express elevated levels of OA-related cytokines [23, 67]. Beyond the impact of post-injury inflammation responses on cartilage and subchondral bone, a direct correlation between gut microbiota and development of injury-induced OA has been previously demonstrated. Significantly, in animal models induced by destabilized medial meniscus (DMM) surgery, the severity of PTOA was reduced in the germ-free situation compared to specific pathogen-free (SPF) mice, providing evidence for a role of the gut microbiota in PTOA pathogenesis [13]. These findings have illustrated the complexity of the inflammatory response to joint injury and gut microbiota is thought to be involved in PTOA progression.

### Other OA risk factors

Other OA-relevant risk factors, such as smoking and alcohol consumption, are also identified as a perturbation on gut microbiota. It is wildly accepted that smoking and alcohol contribute to the unfavorable changes of the integrity of gastrointestinal barrier. Also, alcohol causes the depletion of anti-inflammatory bacteria, eventually resulting in intestinal damages [68]. Besides, in a population-based cross-sectional study, Lee et al. built the link between gut microbiota composition and current smokers [69]. Still, the connection between these OA-relevant risk factors, OA, and gut microbiota has not been demonstrated yet.

## Gut microbiota modulation as treatment of OA

Considering the growing global prevalence of OA, effective disease-modifying therapeutic strategy for relieving symptoms and slowing down OA progress are greatly needed. In this context, it is feasible to hypothesize that the modulation of gut microbiota by external approaches may influence the progression OA. To date, some lines of evidence indicate that gut microbiota interventions may be realized through probiotics, prebiotics, nutraceuticals, exercise, and fecal microbiota transplantation (FMT).

### Probiotics and prebiotics

Probiotics are composed of live microorganisms, generally lactic acid bacteria, which play a crucial part in the maintenance of healthy gut microbiota homeostasis by promoting the production of antimicrobial substances and immunoglobulins and inhibiting the production of bacterial toxins [70]. Recent observations gave rise to hopes that probiotics would provide beneficial treatment strategy in OA. To date, the most convincing evidence is a randomized double-blind placebo-controlled trial performed by Lei et al., in which a total of 537 patients with knee OA were enrolled and randomized to receive skimmed milk containing either *Lactobacillus casei Shirota* (LcS) or placebo daily for 6 months [14]. After 6 months of treatment, clinical outcomes assessed by WOMAC (Western Ontario and McMaster Universities Osteoarthritis Index) and VAS (visual analog scale) scores were significantly reduced in the LcS group compared to the placebo group. Moreover, systematic inflammation assessed by the serum high-sensitivity C-reactive protein (hs-CRP) was also significantly lower in patients receiving LcS than placebo. This study indicates that LcS consumption could serve as a novel therapeutic option in OA management, improving clinical outcomes probably through reducing serum hs-CRP levels. Another study in experimental rodent model of OA found that oral administration of LcS (2 × 1010 cfu/kg, 500 mg/kg) together with glucosamine (Gln; 250 mg/kg) and type II collagen (CII; 250 mg/kg) is effective to reduce pain compared to the control group, while LcS or Gln or CII/Gln alone failed to improve clinical outcome. This study indicated the synergistic effects of LcS/Gln/CII, while the exact weight of each individual treatment is hard to assess and the sample number is insufficient (*n* = 8~9 in each group) [15]. Sim et al. found that oral administration of tyndallized *Clostridium butyricum* effectively preserved articular cartilage and synovial membrane and significantly reduced the amount of fibrous tissue, serum concentration of various inflammatory mediators, and bone metabolism markers in a monosodium iodoacetate-induced (MIA) OA rat model [16]. In the same OA model, Kwon et al. claimed that dietary supplements of probiotic complex (12.5 mg/rat) combined with rosavin (100 mg/rat) and zinc (20 mg/rat) to MIA rats (weighted from 140 to 230 g) slowed down OA development through the inhibition of proinflammatory cytokines and cartilage destruction [17]. However, this evidence is far from convincing due to the limited sample number (*n* = 3) and observation period (13 days). Also, the results were over-interpreted because only pain assessment reached statistical significance while morphology assessments were not significant and the evidence supporting that the combination treatment inhibited inflammation was based on in vitro chondrocyte experiments.

Prebiotics also is selectively utilized by host microorganisms and is able to stimulate the growth of different beneficial bacteria in the gastrointestinal tract [71, 72]. Schott et al. provided evidence that supplementation with oligofructose (OFS) prebiotic fiber, a non-digestible plant-derived carbohydrate, fermented by gut microbiota in the colon, could reverse the adverse effects of high-fat diets on gut microbial composition by increasing the abundance of *Bifidobacterium*, a species with anti-inflammatory properties, resulting in the reduction of systemic and knee joint inflammation, the preservation of articular cartilage, and the suppression of obesity-induced joint structural changes in the context of injury [18]. These findings indicated that prebiotic manipulation of the gut microbiota is a potentially novel candidate therapeutic strategy to treat OA.

### Nutraceuticals

The clinical effects of glucosamine sulfate (GS) and chondroitin sulfate (CS) on OA management is still controversial, even some systematic reviews show their symptomatic and structure-modifying effects contributed by the anti-inflammatory effects on cartilage [73]. In the 2019 guidelines, low-dose, short-term pharmaceutical-grade glucosamine and chondroitin sulfate are recommended by the European Society for Clinical and Economic Aspects of Osteoporosis, Osteoarthritis and Musculoskeletal Diseases (ESCEO) whereas Osteoarthritis Research Society International (OARSI) strongly recommends against their use (including all glucosamine and chondroitin formulations) [74].

Oral supplements of GS and CS have limited intestinal absorption and hence the effects of these supplements on the gut microbiome are of great interest. The systematic review performed by Shmagel et al. summarized the evidence supporting the effects of GS or CS on gut microbial composition that CS increased abundance of genus *Bacteroides*, while the evidence of GC on gut microbiome is limited [75]. Liu et al. found that CS supplementation was associated with increased *Bacteroides acidifaciens*, reduced inflammatory *Proteobacteria*, lower serum LPS levels, and increased level of fecal total SCFA and butyrate in the stressed mice, illustrating that the anti-inflammatory and gut-protective effects of CS supplementation may depend on the specific bacterial species [76]. In particular, *Bacteroides* species account for more than 10% of all human gut microbiota and are primarily responsible for degrading CS, which could help to explain the efficacy of CS as a potential therapeutic drug in OA treatment [77]. Similarly, Coulson et al. demonstrated that oral supplementation with green-lipped mussel extract or glucosamine sulfate have symptom-modulating effects on OA patients by regulating the composition, metabolic, and immunological activities of gut microbiota [19]. Furthermore, GS and CS are also significant components of intestinal mucin, acting as a defense barrier between gut flora and the intestinal wall and potentially affecting gut permeability and intestinal immune responses [78, 79]. Advanced understanding of the biological action of GS and CS on shaping gut microbiome provides a novel insight into their potential mechanisms in OA management, while the convincing evidence to link GS/CS, gut microbiota, and OA is still needed.

### Exercise

The evidence of exercise therapy has indicated the therapeutic benefits by reducing pain and improving functioning for individuals with OA [80]. Beyond the changes of mechanical loading on cartilage and subchondral bone, exercise may modulate the composition, functions, and metabolites of gut microbiota to exert possible benefits for the host [81]. Several mechanisms are involved in the health-promoting effects of exercise, including the improvement of the *Bacteroidetes*/*Firmicutes* ratio, the alteration of bile acid profile, the increased production of SCFAs, the suppression of TLR signaling pathway, the mediation of mucosal immunity via production of immunoglobulin A (IgA), reduced intestinal transit time, and the activation of the HPA axis [82].

Exercise independently alters the composition and functional capacity of the gut microbiota or together with dietary supplements. Munukka et al. showed that taxonomic shifts included an enriched abundance of *Akkermansia* and a decrease in *Proteobacteria* after 6-week exercise among previously sedentary overweight women [83]. In addition, Jacob et al. performed a study to explore the effects of exercise on gut microbiota in lean and obese participants with multiple-day dietary controls, revealing that exercise increased the abundance of *Faecalibacterium* species and the concentrations of acetate, butyrate, and SCFAs only in lean subjects [84]. These findings suggest that exercise-induced changes in the human gut microbiota are largely dependent on obesity status. Furthermore, in response to exercise, the gut microbiota of lean individuals may be more responsive than that of obese or overweight individuals. Several studies also indicated that exercise might also be associated with diet determining the microbial biodiversity of the gut. Clarke et al. was the first to find that exercise increases gut microbial diversity that was positively correlated with dietary protein consumption in the athlete group [85].

In spite of the evidence supporting that exercise benefits host by the underlying involvement of gut microbiota, the finding regarding this effects upon the mechanism of exercise in OA patients, so far, is scarce. Rios et al. showed that prebiotic fiber supplementation, aerobic exercise, and the combination of these two interventions completely prevented the development of OA-like knee joint damage in a high-fat/high-sucrose diet-induced rat model of obesity [20]. In particular, the combination of the two interventions resulted in an increased relative abundance of *Bifidobacterium* and *Roseburia* negatively associated with knee joint damage induced by diet-induced obesity while a decrease in *Clostridium leptum* (cluster IV) and *Akkemansia muciniphila* which exhibited a positive association with joint damage. Future studies should be specifically designed to clarify whether exercise may positively influence this putative gut-joint axis and to investigate the degree to which exercise rebalance gut dysbiosis to influence OA development.

### Fecal microbiota transplantation (FMT)

FMT is a manipulation aimed to treat diseases associated with gut microbiota by transferring of feces from a healthy donor into the distal gastrointestinal tract of a recipient patient [86]. Promising findings suggest that this method has already emerged as a successful treatment against *Clostridium difficile* infection [87] and is currently being demonstrated as a potential therapeutic option of inflammatory bowel disease (IBD) [88]. Also, emerging evidence demonstrates the promising application of FMT in the management of OA. Huang et al. designed a study to collect fecal samples from human healthy controls, knee OA without metabolic syndrome, and knee OA with metabolic syndrome groups, then transplant pooled samples into germ-free OA mice induced by meniscal/ligamentous injury (MLI). Interestingly, only the microbiota transplantation from the knee OA with metabolic syndrome and MLI resulted in an increase in the severity of OA, which was also consistently associated with elevated inflammatory biomarkers and gut permeability [21]. These findings support that an adverse microbiome would exacerbate the histopathological severity of OA induced by joint injury in a murine model. The study is a paradigm for the application of FMT in the investigation of OA pathogenesis and also give promise to the manipulation of gut microbiota in OA management, while more convincing studies are needed due to the limited sample number (*n* = 9).

## Concluding remarks and future perspectives

OA represents a primary public health problem, which is a major source of pain, disability, and socioeconomic cost worldwide. Therefore, interpreting the potential mechanisms of OA pathogenesis is essential for developing novel prevention and disease-modifying therapeutic interventions.

In this narrative review, we summarized the evidence supporting the hypothesis of “gut-joint axis” and the interaction between gut microbiota and the OA-relevant factors and provided the reasonable speculations of the promising manipulation of gut microbiota in OA management. The evidence of the gut microbiota involvements upon the mechanisms of the risk factors, such as obesity and metabolic syndrome and joint injury, is more convincing compared to the others. Given that the intervention on gut microbiota to investigate its role in OA is scarce so far, the connections between gut microbiota and OA risk factors are still inconclusive. As a result, we call for the more convincing human longitudinal studies based on microbiota manipulation. Moreover, it is important to point out that this systematic low-degree inflammation induced by the disturbed gut microbiota is not specific for OA but rather favors the emergence of several potential diseases, such as metabolic syndrome and cardiovascular diseases. In this context, we speculate that the influence of gut microbiota on the OA subtypes may be different. Indeed, most of evidence is based on the Met-OA model, suggesting the potential interaction between gut microbiota, OA, and also other diseases. This adds another layer of complexity to the mechanisms of gut microbiota.

The detailed mechanisms of “gut-joint axis” remain reclusive. It is likely that gut microbiota influences joint by regulating inflammation and metabolism, while the gap between cartilage metabolism and gut microbiota still exists. In the context, multiple omics, such as metabonomic and transcriptomics, are helpful to link specific metabolites, genes, or signaling pathways contributing to the regulation of gut microbiota to decode this intricate matter in a molecular resolution. Also, single-cell technique is promising to reveal the relevance of distinct cell subgroups to gut microbiota. Besides the mechanism investigations, existing evidence also suggests the possibility of novel biomarkers related to inflammation and gut dysbiosis that are able to predict OA progression and monitor the efficacy of therapeutic intervention.

## Data Availability

Not applicable.

## Abbreviations

OA: Osteoarthritis
AGEs: Advanced glycation end products
SCFA: Short-chain fatty acids
IGF1: Insulin-like growth factor 1
GDF5: Growth differentiation factor 5
VDR: Vitamin D receptor
GWAS: Genome-wide association analysis
PUFA: Polyunsaturated fatty acid
AA: Arachidonic acid
DHA: Docosahexaenoic acid
LPS: Lipopolysaccharide
PGN: Peptidoglycan
LBP: LPS-binding protein
HPA: Hypothalamic-pituitary axis
NTS: Nucleus tractus solitarus
SCN: Suprachiasmatic nuclei
ACL: Anterior cruciate ligament
DAMPs: Damage-associated molecular patterns
TLRs: Toll-like receptors
DMM: Destabilized medial meniscus
SPF: Specific pathogen-free
LcS: *Lactobacillus casei Shirota*
Gln: Glucosamine
OFS: Oligofructose
GS: Glucosamine sulfate
CS: Chondroitin sulfate
FMT: Fecal microbiota transplantation
IBD: Inflammatory bowel disease
